# Neural Correlates Associated with Successful Working Memory Performance in Older Adults as Revealed by Spatial ICA

**DOI:** 10.1371/journal.pone.0099250

**Published:** 2014-06-09

**Authors:** Emi Saliasi, Linda Geerligs, Monicque M. Lorist, Natasha M. Maurits

**Affiliations:** 1 Department of Neurology, University Medical Center Groningen, University of Groningen, Groningen, The Netherlands; 2 Department of Experimental Psychology, University of Groningen, Groningen, The Netherlands; 3 BCN-NeuroImaging Center, University Medical Center Groningen, University of Groningen, Groningen, the Netherlands; University Children's Hospital Tuebingen, Germany

## Abstract

To investigate which neural correlates are associated with successful working memory performance, fMRI was recorded in healthy younger and older adults during performance on an n-back task with varying task demands. To identify functional networks supporting working memory processes, we used independent component analysis (ICA) decomposition of the fMRI data. Compared to younger adults, older adults showed a larger neural (BOLD) response in the more complex (2-back) than in the baseline (0-back) task condition, in the ventral lateral prefrontal cortex (VLPFC) and in the right fronto-parietal network (FPN). Our results indicated that a higher BOLD response in the VLPFC was associated with increased performance accuracy in older adults, in both the baseline and the more complex task condition. This ‘BOLD-performance’ relationship suggests that the neural correlates linked with successful performance in the older adults are not uniquely related to specific working memory processes present in the complex but not in the baseline task condition. Furthermore, the selective presence of this relationship in older but not in younger adults suggests that increased neural activity in the VLPFC serves a compensatory role in the aging brain which benefits task performance in the elderly.

## Introduction

Healthy aging is associated with a decline in working memory performance [1] (see for reviews [2]–[4]), which is particularly apparent during complex task conditions. Working memory is defined as a set of cognitive functions involved in the active maintenance and manipulation of information over a brief period of time [5]. Adequate performance on working memory tasks is supported by several, widespread brain regions, such as the bilateral dorsal lateral and ventral lateral prefrontal cortex (DLPFC and VLPFC, respectively), supplementary motor area (SMA), premotor cortex, and posterior parietal cortex, including the inferior parietal lobule (see for a meta-analysis [6]). These regions have been associated with specific working memory processes, such as the manipulation of information (DLPFC [7], [8]), the active maintenance of information (e.g., VLPFC [9]) and the short-term storage of information (inferior parietal lobule [10]). In addition several of these frontal and parietal regions have been associated with more general cognitive control functions, such as the ability to maintain task-relevant goals or task sets in working memory or to orient the focus of attention to the external environment [11], [12]. It has been hypothesized that working memory and cognitive control are not only anatomically but also functionally interdependent. For example, the active maintenance and updating of task-relevant representations in working memory is important for cognitive control processes, such as response selection under interference [13].

Older and younger adults differ in the pattern of brain activation elicited during performance on working memory and related tasks and these age-related differences in brain activation are dependent on the level of task demands. Results from studies implementing a parametrical increase in task load, suggest that older adults reach maximal levels of brain activation at lower task demands than younger adults [14]. As a consequence younger adults show enhanced neural activation in more relative to less demanding task conditions [15], [16], while older adults show either a decrease or a less pronounced increase in activation with increasing task load, mainly in the bilateral DLPFC and/or the VLPFC [17]–[22]. The difference in load-dependent activation patterns in younger and older adults is explained in the compensation-related utilization of neural circuits hypothesis (CRUNCH [23]). The CRUNCH model explicitly postulates that neural involvement is dependent on task demands. According to CRUNCH, older adults engage more cognitive resources than younger adults during ‘easier’ task conditions, possibly to counteract neurobiological declines associated with aging. With increasing task demands, however, these neural resources reach their maximum and brain activation and performance levels decline in older adults [24].

Moreover, effects of aging on activation in brain regions supporting working memory vary as a function of performance level. For older adults, more efficient task performance was found to be associated with higher neural activation [20], [25]. In contrast, reduced or comparable neural activation observed during more complex relative to less demanding task conditions seems to be a feature of low performing older adults [21], particularly in regions such as the DLPFC [8]. Noteworthy is that not only decreased, but also increased activation has been observed in low performing older adults in regions of the right lateral PFC, such as the right DLPFC and the right rostral lateral PFC (RLPFC [26]).

Although generally there is consensus that decreased neural activation in older adults might be related to cognitive decline, the interpretation of increased activation is less clear (see for a review [24]). For example, enhanced bilateral prefrontal activation in older adults in tasks eliciting unilateral activation in young adults, accompanied by successful task performance has been interpreted as a form of neural compensation [3], also referred to as Hemispheric Asymmetry Reduction in Older Adults or ‘HAROLD’ [27], [28] and Grady et al. (1994) suggested that older adults recruited frontal areas to compensate for decreased sensory processing in the occipitotemporal brain regions as described in the Posterior-Anterior Shift in Aging or ‘PASA’ model [29]. Note that these models do not explicitly take into account that neural activation patterns might depend on task demands. On the other hand, increased prefrontal activation, associated with lower task performance was argued to reflect less efficient recruitment of neural resources [30]–[32]. Thus enhanced (prefrontal) activation in the aging brain might either reflect neural compensation, inefficient recruitment of neural resources, or both. Here, we aimed to clarify the association between neural activation and behavioral performance levels in older adults during performance on a working memory task.

The majority of studies so far, have focused on age-related effects in specific brain areas. However, specialized brain regions are organized into integrated functional networks to support cognitive functions [33]. Brain regions forming one functional network seem to be engaged by similar cognitive tasks [34]–[36]. In the present study we specifically investigated the association between behavioral performance in older adults and the activation pattern in functional networks engaged during working memory tasks.

In order to do so, we investigated the effects of aging on functional networks supporting working memory in older adults, during performance on an n-back task (0-back, 1-back and 2-back). To examine working memory-related processes, we focussed on the comparison of behavioral effects and neural activation obtained in the baseline (0-back) and in the complex (2-back) task condition. Working memory-related functional networks were assessed by means of spatial independent component analysis (ICA) of the fMRI data. ICA is a data-driven method that organizes brain regions with a similar time course into spatially independent patterns of blood oxygenation level dependent (BOLD) signal, which are represented in independent components [37], [38]. Brain regions grouped in an independent component can be considered as a functional network. Hence, ICA enables us to investigate how activity in these networks is modulated during performance on the baseline and the complex task condition. In addition, this approach allows us to determine whether the activation associated with specific working memory related components differs between younger and older adults and whether it is significantly associated with performance level. As mentioned above, the functional significance of altered brain activity in older adults is not fully understood. In this study we aim to elucidate the neural correlates associated with successful working memory task performance in older adults. We expect variability in performance among the elderly; while most elderly will show a decline in performance compared to young adults, some older adults will exhibit successful working memory task performance. To understand how these elderly maintain high performance, we will specifically study brain regions that are part of functional networks previously associated with working memory. In line with compensation theories of aging, we expect that older adults will show enhanced activation in working-memory-related functional networks. In addition, we hypothesize that activity in functional networks will be influenced by task demands (as is predicted by the CRUNCH model [23]). In line with this we expect that older adults will show enhanced activation especially in low demanding task conditions. Moreover, enhanced activation in these working memory-related networks is expected to be associated with more successful working memory task performance in older adults [27], [39], [40]. At high demanding task conditions, we expect that this compensation mechanism will break down and that both performance and brain activation will decline in older adults.

## Materials and Methods

The ethics committee of the University Medical Center Groningen, the Netherlands, approved the current study. The subjects who participated in the study were treated according to APA ethical. All participants provided written informed consent to participate in this study.

### Participants

Forty healthy younger adults (mean age 20.6 years; range 18–26 years; 20 males) and forty healthy older adults (mean age 65.1 years; range 59–74 years; 24 males) participated in this study. All participants were right handed and had normal or corrected to normal visual acuity. Exclusion criteria were a history of neurological, psychiatric or vascular disease and use of any psychotropic or hypertensive medications. Data from one younger and one older participant was lost due to technical problems. In addition, one older participant was excluded due to brain abnormalities discovered in the anatomical scan.

### Neuropsychological testing

To verify normal cognitive functioning, all participants were tested on a neuropsychological test battery, consisting of the Mini Mental State Examination (MMSE [41]), the Hospital Anxiety and Depression Scale (HADS [42]), visual-motor sequencing (trail-making test A and B), phonemic fluency (words beginning with the letter “S” and “F”), semantic fluency (professions and animals), Stroop task, working memory (digit span test forward and backward and digit symbol coding test), immediate and delayed recall, as well as recognition (15 words test). An estimation of the intelligence quotient (IQ) was obtained through the Dutch Adult Reading test, the Dutch version of the National Adult Reading Test (NART [43]) and the WAIS-matrix reasoning test [44]. In addition, participants performed a simple reaction time test, during which they were required to press a response button as fast as possible whenever a red dot appeared on the screen. The red dot appeared for 300 ms and inter trial intervals (ITI) varied randomly between 2000 and 6000 ms. Demographic and neuropsychological test results are presented in Table 1, for each age group, for participants included in the final statistical analysis.

**Table 1:** Demographics and neuropsychological test results.

		Young	Old	t-test
	Total number*	38	37	
	Gender, male/female	20/18	24/13	
	Age, years (mean(SD))	20.8(1.9)	64.8(3.5)	
	MMSE^1^	29.5(0.6)	28.5(1.2)	4.2
	NLV IQ^2^	105.4(5.6)	112.1(10.3)	−3.5
	WAIS IQ	111.6(10.6)	110(9.8)	0.7
	HADS anxiety	3.7(2.5)	3.1(2.5)	1.1
	HADS depression	1.8(1.9)	1.9(2.2)	−0.3
**Visual-motor sequencing**				
	Trail making A^2^	26.9(7.6)	37.5(10.8)	−4.9
	Trail making B^2^	55.7(19)	82.4(37.6)	−3.9
**Phonemic fluency**				
	Letter 's'	16.3(5.9)	15.4(6)	−0.7
	Letter 'f'	7(3.9)	7.4(4.1)	−0.4
**Semantic fluency**				
	Professions	20.1(4.9)	20.7(6.4)	−0.5
	Animals	27.2(5.4)	25.4(7.2)	1.2
**Stroop task**				
	Words^2^	41.1(6.2)	47.5(7.3)	−4.1
	Colors^2^	52.4(9.2)	59(14)	−3.2
	Word-Color^2^	75.9(14)	92(17.7)	−4.4
**Memory tasks**				
	15 words test Direct recall^1^	56(7.5)	44.6(8.9)	6
	15 words test Delayed recall^1^	12.1(2.1)	9.1(3)	4.9
	15 words test Recognition^1^	29.7(0.5)	28.3(2.1)	3.7
	WAIS number Forwards	10(2.1)	9.2(13.3)	1.7
	WAIS number Backwards^1^	8(2.7)	6.8(2)	2.2
	WAIS symbol-number substitution^1^	85.4(13)	67.4(13.3)	5.9
**Simple reaction time task** 2		232.8(17.3)	251(28.1)	−3.4

*Neuropsychological data from one young adult was not recorded;

1 Young > Old;

2 Old > Young; t-test =  independent two samples t-test; MMSE  =  Mini Mental State Examination, NLV  =  Dutch Adult Reading test, WAIS matrix  =  matrix reasoning subtest of the Wechsler Adult Intelligence Scale.

### Cognitive task

**Figure 1:**
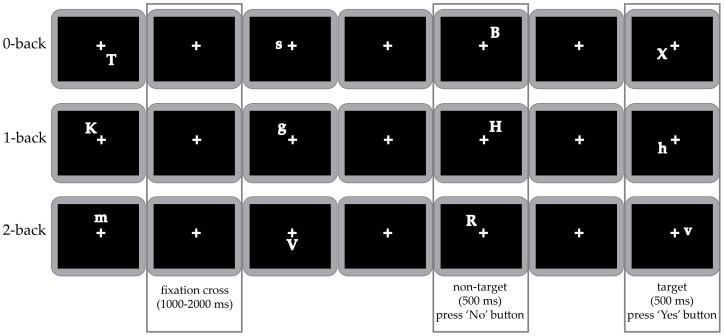
Schematic diagram of the n-back task, separately for each task load. Details about timing for stimulus and the fixation cross are included.

Working memory was tested using an n-back task. Each block started with the presentation of task instructions, followed by a fixation cross, which remained on screen throughout the task presentation. Then a continuous stream of letters was presented in different locations around the fixation cross; one letter per frame, for 500 ms each. The time interval between letter frames varied randomly between 1000 and 2000 ms. Each letter was randomly positioned in one of 8 possible locations (horizontal X axis, vertical Y axis and the lower and upper position of both diagonals; see Figure 1). The distance between the participant and the projection screen was approximately 90 cm. The visual angle between the participant and the stimulus was approximately 3.6 degrees.

The n-back task had three load conditions; 0-back, 1-back and 2-back. The visual input was identical in all loads and the conditions could only be differentiated through the instructions received. In the 0-back load condition, the target was the letter ‘x’. In the 1-back load condition, the target was any letter identical to the letter immediately preceding it. In the 2-back load condition, the target was any letter identical to the letter presented two trials ago. Each load condition was presented twice, resulting in a total of six task blocks, with 100 trials each. In each block, targets occurred randomly in 50% of the trials. The order of the task loads was semi-randomized between participants.

The letters were chosen from a set of 18 consonants derived from the Dutch alphabet (all consonants except the letters Q, Y and J). Letters were displayed in white in a 40 point Arial font on a black background and were randomly presented either in upper-case (50%) or lower-case (50%). Participants were instructed to ignore the case of the consonant and to focus on its identity. Responses were given manually by pressing one of two buttons on an air pressure button box device. For half of the participants a right index finger response was required for targets and a right middle finger response was required for non-targets. For the other half of the participants index and middle finger responses were reversed. E-Prime 1.2 software (Psychology Software Tools Inc., Pittsburgh USA) was used for stimulus generation and response registration.

### Image acquisition

Functional MRI scans were obtained with a 3T MR scanner (Philips Medical Systems, Best, Netherlands), with echo planar imaging (EPI) capability and an eight channel SENSE head coil. Functional images were obtained in one run with the following pulse sequence parameter settings: single shot EPI; 37 slices; slice thickness 3.5 mm; no gap; field of view 224 mm; matrix scan size 64 by 62; transverse slice orientation; repetition time (TR) 2000 ms; echo time (TE) 30 ms; minimal temporal slice timing 1836 ms; flip angle 70°. A 3-D T1-weighted anatomical scan of the entire brain was obtained for each participant using the following pulse sequence parameters: field of view 256 mm; matrix scan size 256 by 256; 170 slices; slice thickness 1 mm; transverse slice orientation; TE 3.6 ms; TR 9 ms; flip angle 8°.

### Procedure

Participants visited the laboratory twice. During the first visit, demographical and health questionnaires were filled out, neuropsychological tests were administered and the experimental task was practiced. Participants practiced each of the 3 task conditions, until the task was fully understood. Throughout the practice session, participants received feedback on their performance levels after each task condition. During the second visit, participants practiced again after which they performed the six blocks (two blocks for each condition, each consisting of approximately 200 scans) of the n-back task while fMRI was recorded in one run. In addition, an anatomical scan was obtained.

### Data analysis

#### Behavioural data analysis

Responses faster than 200 ms and slower than 1500 ms were considered as incorrect. Performance accuracy was calculated as the rate of correct responses given by the participant in each load condition, compared to the total number of trials in that load condition. The median reaction time (RT) for correct responses was calculated. One older participant was excluded based on below chance performance (less than 50% in the 1-back and the 2-back load conditions).

#### Working memory performance index

To investigate working memory-related processes, we focused on the difference in RT and accuracy scores between the 2-back and the 0-back load conditions. The 0-back and 2-back load conditions both contained identical visual information and differed only in terms of maintenance load and temporal ordering demands [45]. More specifically, in the 0-back load condition, participants were required to remember one letter (the letter ‘x’). In the 2-back load condition, participants were required to maintain two letters in working memory. These letters changed continuously, requiring constant updating of information in working memory [20]. Moreover, in the 2-back load condition participants needed to keep track of the temporal order in which these letters were presented. By focusing on the differences between the two load conditions, effects of motor-related activity and perceptual processing common to both load conditions are removed. In contrast, working memory-related effects dissociating both conditions, such as active maintenance, working memory updating and temporal order information, remain. The 1-back load condition also requires constant updating of information, but in this condition keeping track of temporal order information is not required. The inclusion of the 1-back condition might provide a more detailed analysis of the sub-processes involved in working memory. However, we had no prior hypothesis about functional networks supporting these separate sub-processes within the working memory system.

In this study, specific working memory performance indices were constructed using two dimensions of processing efficiency (speed and accuracy). First, the relative increase in RT from the baseline load condition (0-back) to the most complex load condition (2-back) was calculated for each individual ((2-back _RT_ – 0-back _RT_)/0-back _RT_). Second, the relative decrease in accuracy scores between both load conditions was calculated for each participant ((0-back _accuracy_ – 2-back _accuracy_)/0-back _accuracy_). For both variables, higher values reflect less efficient performance. The relative increase in RT will be referred to as *speed cost* and the relative decrease in accuracy scores will be called *accuracy cost*.

### fMRI image analysis

#### Preprocessing

Pre-processing of neuroimaging data was performed using the statistical parametric mapping software package (SPM 8; http://www.fil.ion.ucl.ac.uk/spm/software). First, functional images were motion-corrected and coregistered to the anatomical scan. The coregistration step was inspected visually and adjusted if necessary. Second, bias regularization was applied to the anatomical scan to reduce smooth signal intensity variations caused by field inhomogeneities. Subsequently, bias regularization was applied to the functional images in the following manner: 1) bias regularization was performed on the first and the last functional image, 2) an average correction factor was calculated for each voxel based on these two corrections and 3) this average correction factor was applied to all functional images. Third, the individual T1-weighted anatomical images were segmented using a segmentation algorithm to produce grey matter (GM), white matter (WM) and cerebral spinal fluid (CSF) segmentations, in the native space of the T1 scans. This segmentation algorithm provided by SPM8 includes unique tissue probability maps. Fourth, Diffeomorphic Anatomical Registration Exponentiated Lie algebra (DARTEL) was used to create a customized anatomical template from the specific templates of all our participants (younger and older adults together, excluding the older participant with a brain abnormality). DARTEL optimizes inter-participant alignment [46]. In the final pre-processing step, the data were smoothed with an 8 mm full-width half maximum (FWHM) Gaussian kernel. Data from one younger participant was excluded from further analysis due to excessive movement.

#### Independent component analysis (ICA)

To identify neural activity patterns related to working memory, a spatial ICA decomposition of the pre-processed fMRI data from all participants was performed using the Group ICA of fMRI Toolbox (GIFT [47]). We estimated 50 independent components (ICs) using the infomax algorithm. The minimum description length (MDL) criteria implemented in GIFT, estimated 40 ICs. After visual inspection, we concluded that 40 ICs were not sufficient to separate movement artefacts from task-related activation in our dataset. Hence, an additional 10 ICs were estimated, this number was chosen to be in the middle of the participant with the lowest (30) and the participant with the highest (73) number of estimated ICs. The most stable estimation of the components was achieved by re-running the ICA analysis 10 times, using the ICASSO option in GIFT. The ICs' time courses and spatial maps were standardized using z-scores. The group ICA resulted in 1) 50 ICs and their associated time courses on the group level (based on data from all participants) and 2) 50 ICs and their associated time courses on the individual level, obtained through back projection.

To determine the association between the ICs and the n-back task conditions, a temporal regression was performed on each of the time courses associated with the 50 ICs and the General Linear Model (GLM) design matrix, separately for each participant. The GLM design matrix was derived by modeling the task events (onsets), as well as the movement parameters derived from the realignment step and their first derivatives, each convolved with three basis functions of the hemodynamic response function (HRF): the canonical HRF, its time derivative and its dispersion derivative. Since aging has been associated with changes in the microvasculature in the brain which in turn can affect the neurovascular coupling [48], we used these three basis functions to account for possible age-related variability in the shape of the hemodynamic response function (HRF) [49]. The following columns of the GLM design matrix were entered as separate regressors; the onsets of the three task conditions (all events (approximately 200 scans per task condition) in the 0-back, 1-back and 2-back condition blocks), the six movement parameters and their first derivatives, and the constant of the GLM regression model. Since an important goal of the current study was to investigate the association between performance level and working memory-related activation patterns, the onsets of incorrect responses were *not* excluded.

The temporal regression between the IC time course and the GLM design matrix resulted in three beta weights (one for each basis function) for each participant in each of the three task conditions. To obtain a single beta weight per condition per subject, the ‘*area under the curve*’ was calculated for the reconstructed BOLD response ((beta weight_HRF_*HRF + beta weight_time derivative_*time derivative + beta weight_dispersion derivative_*dispersion derivative)/sum of HRF; see [50] for a similar approach). The resulting beta weights represent the task-related modulation of an IC relative to the rest blocks, comparable to the GLM model fit performed on fMRI data [37]. As such, the beta weights will be referred to as BOLD activation of a specific IC in each task condition.

#### ICs related to working memory processes

Various studies have used the n-back task to study brain activity underlying working memory (see for a thorough meta-analysis [6]), hence neural correlates supporting working memory functions during this task are well-known. This study focuses specifically on the relationship between performance level and working memory-related neural correlates. Therefore, based on the extended literature [6], [51], ICs containing spatial activation maps previously associated with working memory were selected. We selected these ICs visually. We did not select ICs on the basis of the relation between their time courses and the design matrix, as in our study this method cannot be applied for several reasons. First, the selection of task-related components would require us to a-priori choose one or multiple specific task conditions on which this selection should be based, which might bias the results. Second, the choice of a specific correlation threshold for components that should or should not be included in the analysis is a difficult matter, for which no pre-defined criteria are available. Finally, using a selection criterion that is independent from the data that will be analyzed is preferred.

**Figure 2:**
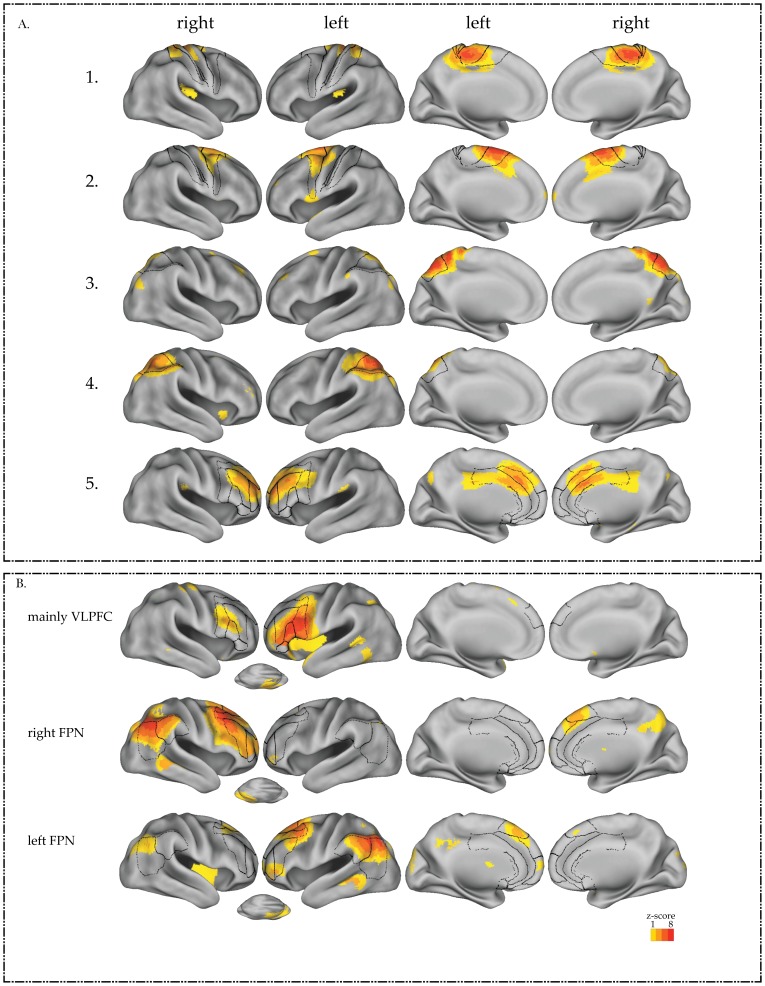
**A.** Independent components (ICs) previously associated with working memory performance. Left two columns: lateral view; Right two columns: medial view. From top to bottom: 1) IC representing mainly the primary motor cortex, 2) IC representing mainly the bilateral SMA, two ICS representing the parietal cortex, namely 3) the medial superior parietal cortex and 4) the superior parietal cortex and 5) an IC representing the executive function network including among others the anterior cingulate, paracingulate gyrus and the VLPFC. B. Working memory related ICs of interest showing age- and load-dependent modulation. From top to bottom, 1) IC representing mainly areas in the lateral prefrontal cortex, with a predominant focus of activation in the left VLPFC. Brodmann areas belonging to the inferior frontal gyrus (44,45 and 47) and to the DLPFC (9 and 46) are presented in the figure; 2) the right FPN and 3) the left FPN. Brodmann areas belonging to the prefrontal cortex (9,24,32,33,44,45, 46 and 47) and to the parietal cortex (39 and 40) are presented in the figure illustrating the right and left FPN; L = left and R = right. IC loading is projected on an inflated surface rendering of the human brain using the CARET program [70].

Visual selection resulted in 8 ICs, containing mainly the following spatial activation maps 1) the pre SMA, 2) the bilateral SMA, 3) the medial superior parietal lobe, 4) bilateral superior parietal lobes, 5) an executive control network (similar to [52] including among others the anterior cingulate, paracingulate gyrus and the VLPFC, 6) the bilateral VLPFC, more prominent in the left side, 7) the right frontoparietal network (FPN) and 8) the left FPN (Figure 2A and 2B). The names attributed to each of the working memory-related ICs are a description of the spatial map represented in each IC. For these 8 ICs, BOLD activation (beta-weights, see the previous section) in the 2-back load condition was contrasted with BOLD activation in the 0-back load condition. This contrast will be referred to as the *BOLD load effect*.

### Statistical analysis

The BOLD load effect data of the eight ICs was entered in a repeated measures ANOVA (SPSS Inc., Chicago, IL) with component (the eight working memory related ICs) as a within-subject measure factor and age (young and old) as a between-subjects measure factor. ICs that showed a significant interaction between age and BOLD load effect were considered as components of interest and studied in further statistical analysis, following a procedure similar to Kim et al [37]. Results of this main repeated measure ANOVA and of the post-hoc tests performed to identify the ICs of interest are described in the Results section (3.2). To investigate the relation between age, performance and BOLD activation in the components of interest, a linear regression analysis was performed in SPSS in which accuracy cost and speed cost were entered as the dependent variable in separate regression models. The variable age was effect coded and centred, and entered as a predictor. In addition, the BOLD load effect in the components of interest was z-scored, separately for younger and older adults, and entered as a predictor. Interactions between age and the BOLD load effect and their effect on the dependent variable were also investigated, in each regression model. If significant effects (p<.05) were observed for any predictor, additional tests were performed to determine whether the association between the behavioral outcomes and age and/or BOLD activation in each IC were present in the 0-back, in the 2-back or in both load conditions. The percentage of variance explained in each analysis and the corresponding F test statistic are reported. Possible associations between BOLD activation and performance level were assessed by Spearman's rank correlation coefficient. Age-related effects on neuropsychological test scores were analyzed by means of a two-sample t-test (Table 1).

## Results

### Neuropsychological testing

Main demographics and mean scores on each neuropsychological test are presented in Table 1, separately for each age-group. In addition, statistics related to age-related differences on the neuropsychological tests are presented in Table 1. Younger and older adults did not differ in average WAIS matrix IQ scores, HADS scores, verbal fluency scores or WAIS number forwards scores. However, older adults had lower average MMSE scores and higher crystallized intelligence scores (as measured by the NLV IQ score) than young participants. Moreover, older adults were slower in the visual-motor sequencing task, the Stroop task and in a simple reaction time test than young adults. Likewise, older adults had lower scores on the following measures of memory: WAIS digit span backwards and the 15-words test comprising of direct recall, delayed recall and recognition.

### Performance and age

Older adults had a mean accuracy cost of.23 (SD = .12) and a mean speed cost of.47 (.19). Younger adults had a mean accuracy cost of.08 (.04) and a mean speed cost of.31 (.18). Coefficients for the main effects of age on accuracy and speed cost were based on the regression model assessing the effects of age and/or BOLD load effect in the VLPFC component. Older adults had a higher accuracy cost (β age = .147, t(74)  = 7.52, p<.0005; *R*2 = .506, F(3,74)  = 24.3, p<.0005) and speed cost (β age = .167, t(74)  = 4.02, p<.0005; *R*2 = .218, F(3,74)  = 6.6, p<.0005) than younger adults. Additional regression models, performed in each load condition, revealed that older adults had lower accuracy scores in the 2-back load than younger adults (β age = −.129, t(74) = −6.39, p<.0005; *R*2 = .497, F(3,74)  = 23.4, p<.0005). Furthermore, older adults were slower than younger adults in both load conditions (0-back: β age = 115.32, t(74)  = 9.81, p<.0005; *R*2 = .579, F(3,74)  = 32.5, p<.0005 and 2-back: β age = 249.01, t(74)  = 9.71, p<.0005; *R*2 = .578, F(3,74)  = 32.3, p<.0005; see Table 2).

**Table 2:** Behavioral results: mean RTs in milliseconds (ms) and the proportion of accurate responses, for younger and older adults.

	Young				Old	
	0-back	1-back	2-back	0-back	1-back	2-back
**RT** (ms; mean(SD))	473(51)	511(59)	617(98)	589(50)	668(84)	866(125)
**Accuracy** (proportion)	.93(.03)	.90(.05)	.86(.04)	.94(.03)	.90(.04)	.73(.12)

Behavioral outcomes are presented for the 0-back, 1-back and 2-back load condition.

### Components of interest: Age and BOLD load effect

The main repeated measure ANOVA on the BOLD load effects observed in the eight ICs associated with working memory processes, showed a general interaction between age and BOLD load effect (F(7,511) = 4.8, p<.0005). To identify which of these 8 ICs showed an age-related BOLD load effect, additional post-hoc two sample t-tests were performed. These tests revealed that the difference in BOLD activation between the 2-back and the 0-back load condition was larger for older than younger adults in 3 ICs. Namely, the ICs containing mainly the VLPFC (t(73) = 2, p = .049), the right FPN (t(73) = 2.2, p = .035) and the left FPN (t(73) = 4.4, p<.0005).

**Figure 3:**
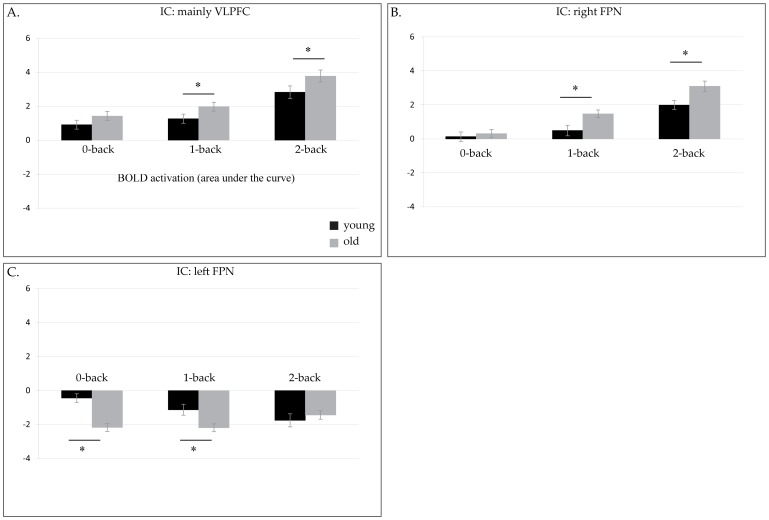
BOLD activation (area under the curve) in A) VLPFC, B) right FPN and C) left FPN, for younger (black) and older adults (grey), in the 0-back, 1-back and 2-back load conditions. Significant differences in BOLD activation between younger and older adults are noted by a star.

After identifying these 3 ICs, we subsequently performed a one-sample t-test in younger and older adults, separately. The purpose of these tests was to investigate whether the BOLD activation in each of the 3 selected ICs differed significantly between the 0-back and the 2-back load condition within each age-group. The one-sample t-test was significant in all 3 ICs, for younger (VLPFC: t(37) = 8.1, p<.0005; right FPN: t(37) =  5.6, p<.0005 and left FPN: t(37) = −4.4, p<.0005) and older adults (VLPFC (t(36) =  10.1, p<.0005), the right FPN (t(36) = 10.2, p<.0005 and the left FPN (t(36) = −2.1, p = .048). For all participants, the BOLD activation in the right FPN and the VLPFC increased with task load. However, the BOLD activation of the left FPN was negatively modulated by the task, as revealed by the negative beta-weights and the positive spatial map of this component (see Figure 2B and 3). In young adults, the BOLD activation in the left FPN became more negative with increasing task demands. To determine whether age modulated the BOLD signal in these 3 ICs of interest in the 0-back, in the 2-back or in both load conditions, subsequent post-hoc independent two sample t-tests were performed. These tests showed that compared to younger adults, older adults had a higher BOLD activation in the VLPFC (t(73) = 3, p = .003) and the right FPN (t(73) = 2.7, p = .008), in the 2-back load condition. In the 0-back load condition, younger and older adults showed similar BOLD activation in the VLPFC and the right FPN. On the other hand, older adults had a more negative BOLD response in the left FPN than younger adults, in the 0-back load condition (t(73) = 5, p<.0005). Younger and older adults had comparable BOLD load effect in the other working memory related ICs.

### Performance and BOLD activation

**Figure 4:**
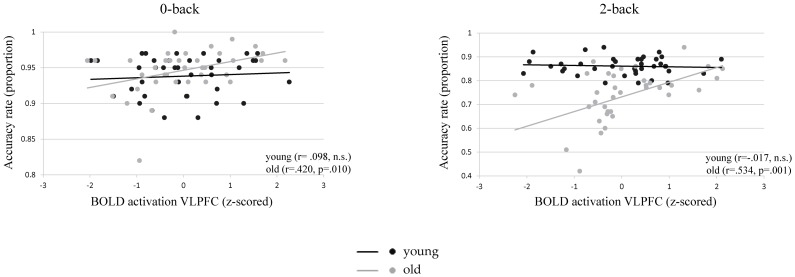
BOLD activation (z-scored) is positively correlated with accuracy rate (proportion) in the 0-back (left) and the 2-back (right) load condition, for older (grey) but not for younger adults (black). Note that the axes have been scaled individually to optimize visualization.

We determined the association between behavioral outcomes (accuracy cost and speed cost) and BOLD load effect in each of the 3 ICs, in separate linear regression models (see section 2.8). The BOLD load effect in the VLPFC was significantly associated with accuracy cost and this association differed between age groups (β _age*load effect VLPFC_ = −.052, t(74)  = −2.63, p = .010). A larger BOLD load effect in the VLPFC was associated with smaller accuracy cost in older adults (β _load effect VLPFC old_ = −.056, t(36)  = −3.04, p = .004; *R*2 = .209, F(1,36)  = 9.3, p<.0005). There was no significant association between accuracy cost and BOLD load effect in younger adults (β _load effect VLPFC younger_ = −.004, t(37)  = −.59, n.s.). To further investigate the association between accuracy rate and BOLD activation in the VLPFC in older adults, separate regression models were performed for each load condition, with accuracy rate as a dependent variable and the mean BOLD activation in the VLPFC component (z-scored, separately for older and younger adults) as a predictor. A larger mean BOLD activation in older adults was associated with higher accuracy rates in both the 0-back (β _BOLD activation VLPFC old_ = .012, t(36)  = 2.51, p = .017; *R*2 = .153, F(1,36)  = 6.4, p = .017) and in the 2-back load condition (β _BOLD activation VLPFC old_ = .061, t(36)  = 3.46, p = .001; *R*2 = .255, F(1,36)  = 12, p = .001; see Figure 4). The correlations between performance accuracy and BOLD activation in the VLPFC in older adults remained significant even after removal of outliers, for both task load conditions.

Moreover, z-statistics [53] were used to investigate whether the correlations between accuracy scores and BOLD activity in the VLPFC differed between younger and older adults. In the 0-back load condition, the ‘accuracy-BOLD’ correlations did not differ significantly between age groups (Spearman's rho _young_ = .098 and Spearman's rho _old_ = .420, z = −1.451, p = .147). In the 2-back load condition, the ‘accuracy-BOLD’ correlations differed between the younger (Spearman's rho = −.017) and the older (Spearman's rho = .534) adults (z = −2.545, p = .011).

A trend towards significance revealed that the BOLD load effect in the right FPN was associated with accuracy cost (β _load effect right FPN_ = −.018, t(74)  = −1.69, p = .095; *R*2 = .423, F(3,74)  = 17.3, p<.0005). Separate linear regression analyses were performed in each load condition, to investigate the association between accuracy and BOLD activation in the right FPN in younger and older adults. A larger BOLD response was related to higher accuracy rates in the 2-back load condition, in both age groups (old: β _BOLD activation right FPN old_ = .057, t(36)  = 3.11, p = .004; *R*2 = .217, F(1,36)  = 9.7, p = .004 and younger (β load effect right FPN younger = .012, t(37)  = 1.92, p = .063; *R*2 = .093, F(1,37)  = 3.69, p = .063). Moreover, a more similar BOLD response between the two load conditions in the left FPN, was associated with a smaller increase in RT in the 2-back relative to the 0-back load condition in younger adults (β _load effect left FPN younger_ = −.079, t(37)  = −3.047, p = .004; *R*2 = .205, F(1,37)  = 9.28, p = .004; referring to β _age*BOLD load effect of the left FPN_ = .093, t(37)  = 2.28, p = .026). We did not observe a significant interaction between speed cost and BOLD load effect in this IC for older adults (β _load effect left FPN old_ = .014, t(36)  = .44, n.s). The association between speed cost in younger adults and BOLD load effect in the left FPN was investigated separately for each load condition. Response speed was not associated with mean BOLD activation in either load condition.

## Discussion

To elucidate the neural correlates associated with successful working memory task performance in young and older adults we used a spatial ICA decomposition of fMRI data collected during n-back task performance. We showed that a higher BOLD signal of the VLPFC is predictive of efficient working memory performance in older adults. Moreover, we observed increased neural activity in the more complex relative to the baseline task condition in older compared to younger adults, in the VLPFC and the right FPN.

Compared to young adults, the elderly showed performance declines in a variety of neuropsychological tests, measuring visuo-motor sequencing, selective attention and inhibition (Stroop task), simple response time, as well as, memory functions. Likewise, we observed that in line with our expectations, older adults generally declined in performance compared to young adults on the n-back task, and that they showed a larger relative increase in RT (speed cost) and a larger relative decrease in accuracy rates (accuracy cost) from the baseline (0-back) to the complex (2-back) condition than younger adults. In the complex task condition participants had to maintain two target letters and associated temporal order information in working memory. This information changed continuously, requiring a constant updating of working memory. In the baseline condition, the target letter remained the same (the letter ‘x’), making this load condition a short-term memory task that requires mainly passive storage and matching. While ‘short-term’ and ‘working’ memory are often confused, short term memory tasks do not necessarily require working memory processes (see [54]). The more pronounced decrease in speed and accuracy in the complex task condition compared to the baseline condition in older adults indicate that ageing indeed affects working memory performance.

In addition to the behavioural indices (i.e. speed and accuracy) we examined neural activity underlying working memory performance in both young and older individuals. In accordance with previous studies we found changes in activity patterns in brain areas implicated in working memory. Moreover, our results showed that variability in performance was related to differences in neural network activity in the older participants. The data revealed that a smaller accuracy cost in older adults was associated with a larger BOLD load effect (increase in BOLD from the 0-back to the 2-back load condition) in the component reflecting mainly the VLPFC. However, such a ‘BOLD-accuracy cost’ association was observed in both the baseline and the complex task condition. Contrary to the behavioural data, this result suggests that the effects of aging are not specific for working memory. The neural mechanisms underlying successful task performance in older adults do not seem uniquely related to specific working memory processes, such as continuous updating of working memory which are especially relevant in the 2-back condition. The load independent age effect on brain activity is in line with the findings of our previous EEG study [55], where we showed that performance efficiency in older adults was negatively related to P3 amplitude in the baseline condition (0-back) as well as to P3 amplitude in the more complex task condition (1-back).

Larger BOLD activation in the VLPFC was also associated with higher accuracy rates in the elderly in both the baseline and complex task condition. The VLPFC, comprising mainly the inferior frontal gyrus (Brodmann areas 44, 45 and 47), has been particularly associated with the active maintenance of verbal information during working memory task performance (BA 44 [40]). The process of active maintenance has been considered as an important subsystem of working memory, responsible for the active rehearsal and storage of information in the absence of external stimuli [56]. In addition, the VLPFC has been found to be activated during the process of comparing a probe (stimulus) with information held online in order to make a judgment on whether the probe is a target or non-target [57]. Higher levels of activation in this brain area in older adults might therefore indicate that maintenance and probe comparison processes are more strongly involved in n-back task performance in older as compared to young participants.

Neuroimaging studies have indeed shown enhanced activity in the VLPFC in response to increased amounts of information maintained in working memory [15], [16]. The positive association between accuracy rates and BOLD activation in the VLPFC in older adults is in line with these findings, and may suggest that optimal performance in the less and the more demanding condition of our task relies on more successful maintenance of verbal information and/or successful ‘stimulus-probe’ comparisons. However, if this is the case, it is not clear why the relation between VLPFC activity and accuracy was observed solely in our older and not in the younger adults, especially since our younger adults were generally more accurate than older adults in the more complex task condition.

N-back task execution relies on a number of complex operations occurring simultaneously in the brain, among which encoding, maintenance and retrieval processes. An alternative interpretation of our findings might be that the BOLD activity measured in the VLPFC represents a summation of simultaneously active sub-processes (see also [45]) instead of the exclusive contribution of the above mentioned working memory related processes. One of these additional processes required during n-back task performance, which has also been associated with VLPFC activation, is cognitive control [58], [59]; see also [60]). Cognitive control is defined as the general ability to actively maintain task-relevant goals or task sets in memory, and to orient the focus of attention on task relevant information [11], [12], all to flexibly adapt behaviour to current task demands. Cognitive control is engaged during both simple and more complex task conditions [61]. Healthy older adults have been found to show deficits in performance especially during a variety of tasks requiring goal-directed cognitive control [61], [62]. The increased BOLD load effect in older compared to younger adults in the component representing the VLPFC, could therefore suggest that older adults engage more cognitive control during task execution in the present study. Following this ‘cognitive control’ framework, the positive ‘BOLD-performance’ relationship may additionally indicate that the ability to engage more controlled processing benefits task performance in older adults.

From the results of the present study no definite conclusion can be drawn regarding the precise processes in which the VLPFC is involved during n-back task execution. Nevertheless, the selective presence of a positive ‘BOLD-performance’ relationship in older adults indicates that the presence of this increase in activation in older adults serves a compensatory role, in line with our expectations. In addition, the presence of this relationship in both less and more demanding task conditions suggests that this compensatory neural mechanism has a general nature and is not uniquely related to sub-processes within the working memory system.

In addition to the age-related modulation of VLPFC activity, the load-dependent activation pattern of younger and older adults differed in two additional ICs representing brain areas associated with the left and the right FPN, respectively. The BOLD response in the right FPN was larger in the complex than in the baseline condition, particularly for older adults. In contrast, the BOLD response in the left FPN was negatively modulated by task load. For older adults, the BOLD response in the left FPN was more negative than for younger adults in the baseline condition, while this pattern was reversed in the complex condition. Moreover, a smaller change in BOLD activation between the two conditions was related to a smaller increase in RT in the complex relative to the baseline condition. In general, verbal working memory tasks have been associated with a prominent left-hemispheric activation in both frontal and parietal regions [40]. The activation pattern we observed in the left and in the right FPN, does not provide support for a verbal versus spatial organization of working memory in the current task, the left FPN was negatively modulated by the current task.

The results of the ICA decomposition, used to identify working memory-related functional networks, showed that the left and the right FPN were represented in separate ICs, indicating that activity in brain areas in the spatial maps of these ICs was differentially modulated by the working memory task. The right FPN was comprised mainly of regions such as the right superior and medial frontal gyrus, the right anterior cingulate, the right inferior parietal lobule and the left cerebellum. The FPN has been found to be active across a variety of tasks requiring controlled processing of information (see for a meta-analysis [51]). In particular, the right FPN has been linked to sustained attention during performance on demanding cognitive tasks [63]–[65] (see for an overview [66], [67]). Sustained attention has been described as the ability to maintain alertness during prolonged time intervals and has been associated with effortful top-down control [68]. Sustained attention is essential during performance on the current n-back task, which requires constant monitoring and processing of the presented stimuli. Compared to younger adults, older adults showed a higher BOLD response in the right FPN during performance on the more complex task condition. This finding suggests that sustained attention was more heavily loaded in older adults. However, despite the higher levels of sustained attention, younger adults outperformed the elderly.

The left FPN consisted mainly of regions such as the left inferior and superior frontal lobe, the left inferior parietal lobule and the right cerebellum. The BOLD response in the left FPN was negative in both task load conditions. This negativity became more pronounced with increasing task demands in younger adults. Results similar to ours have been found in a recent study of Xu and colleagues [38], in which an increase in task complexity was related to an increased negative BOLD response in the left FPN. In their study, activity in the left FPN was argued to reflect interference with top-down control of attention, which should be suppressed. However, their interpretation of the left FPN activity with respect to our findings should be taken with caution. The interpretation of Xu et al. indicates that more activity might benefit task performance. The relationship between BOLD response in this network and the speed cost in our younger adults, however, seems to suggest that the increase in BOLD negativity with task demands does not benefit task performance.

We intended to interpret the identified age-related effects on the modulation of BOLD activity in response to task demands using the CRUNCH model [23]. The CRUNCH model explicitly predicts how age-related differences in neural activation vary with the level of task demand. This model suggests that older adults engage more neural resources than younger adults during easy task conditions, possibly to counteract neurobiological declines in the aging brain. With increasing task difficulty, neural resources reach their maximum and this compensatory mechanism becomes insufficient to counteract age-related decline. Consequently, the brain activation and performance level decrease in older adults in more demanding task conditions [23] (see [24]). Findings from previous studies are in line with the predictions of the CRUNCH model. For instance, a less pronounced load-dependent BOLD response or a decrease in activation in higher compared to lower n-back load conditions in older adults have been reported, in key regions such as the bilateral DLPFC [20], [69] and the bilateral VLPFC [20]. A pattern in which FPN activation in the aging brain, particularly in the prefrontal areas, remains unchanged or decreases with task demands has also been observed during other types of working memory tasks [17], [19].

In the current study however, compared to younger adults, older adults showed increased BOLD activation in the more complex, relative to the baseline condition, in the VLPFC and the right FPN. The load-dependent modulation of BOLD activity presented in Figure 3 suggests that the present findings offer partial support for the CRUNCH model. In line with the expectations of this model, we observed higher BOLD activation in the VLPFC and right FPN in the baseline condition in the elderly compared to the young adults. However, although the higher BOLD activation observed in older compared to younger adults in the more complex task condition seems to suggest that our elderly are well within the limits of their neural resources, their performance levels suggest that they were not able to fully compensate age-related neurobiological decline. Therefore, findings related to BOLD activity in the complex task condition are not entirely in line with other studies [20], [69] that offer support for the CRUNCH model. We argue that differences between our study and other studies may be related to the relative level of working memory load used in the different studies. Whereas we compared the 0-back condition with a 2-back condition, Mattay et al [69] and Nagel et al [20] used an additional level of task difficulty, the 3-back load condition. The increase in BOLD activation with load observed in our study suggests that, according to the CRUNCH model, older adults were still within working memory capacity during performance in the 2-back load condition. It may be that BOLD activation would have decreased at even higher task demands. Remarkable is that, although BOLD activity increased in our study from the 0-back to the 2-back condition and decreased in theirs, the accuracy scores and RTs obtained by our older adults in the 2-back load condition are similar to the behavioral results obtained by older adults in the study of Mattay and colleagues in the 2-back condition. It thus seems that indices of age-related changes in performance and underlying brain activity do not have a one to one relationship.

In addition, differences in effects might be related to differences in the analysis approach used. The above-mentioned studies focussed on activity observed in distinct brain regions, using analysis techniques such as region of interest (ROI) analysis [19], [20] or analysis of variance [17], [69]. Univariate BOLD analysis results in a beta weight representing the activation in each voxel or each specific region, typically for a task condition relative to rest (e.g. baseline). When using ROI analysis, specific anatomical coordinates are generally selected based on findings from previous studies or based on results from contrasts between task conditions of interest. In the current study, however, we did not focus on activation in segregated brain areas. Instead, we used the ICA technique to identify functional brain networks that commonly consist of a subset of brain areas. ICA is a data-driven method that does not require prior specifications of the task conditions. The BOLD activation in each of the identified networks or components of interest reflects weighted synchronous activation from connected regions underlying specific working memory processes. Using the ICA approach, it is not possible to predefine regions reflecting a specific independent component. Therefore, results from a univariate BOLD analysis and ICA are not equivalent and cannot be directly compared to each other. Although, the age-related effects on BOLD activation related to task difficulty as observed in the present study are not fully in line with previous studies, it should be noted, that increased load-dependent activation in ROIs centred on the anterior cingulate, and the bilateral inferior frontal gyrus [25] as well as the premotor cortex and DLPFC [20] has been found previously in high performing older adults. Thus, although our results are not entirely in line with the predictions of the CRUNCH model, they do suggest the presence of functional compensation in the aging brain. Noteworthy is that in the present study the BOLD load effect was affected by aging only in a subset of functional networks previously associated with working memory [6], [51]. Hence, aging seems to selectively affect neural correlates supporting working memory performance.

In summary, in the current study we found age- and performance-related effects on neural activity during performance on a working memory task. Compared to younger adults, older adults had a larger increase in BOLD activation in the VLPFC and the right FPN, in the more complex compared to the baseline task condition. Other regions, such as the SMA and the medial parietal cortex did not show differential BOLD load effects across younger and older adults. Furthermore, our study identified a positive association between VLPFC activity and performance accuracy in older adults. This ‘BOLD-performance’ relationship was observed in both the baseline and the complex task conditions, supporting that the neural mechanisms underlying performance in older adults are not uniquely related to working memory processes such as manipulation or updating of information. The selective presence of this relationship in older adults indicates that this pattern of neural activation is beneficial for task performance and can therefore be regarded as a compensatory mechanism in the aging brain.
