# Correction: Neural Correlates Associated with Successful Working Memory Performance in Older Adults as Revealed by Spatial ICA

**DOI:** 10.1371/journal.pone.0151185

**Published:** 2016-03-03

**Authors:** Emi Saliasi, Linda Geerligs, Monicque M. Lorist, Natasha M. Maurits

There are errors in the fourth and fifth sentences of the Abstract. The correct sentences are: Our results indicated that a higher BOLD response in the VLPFC was associated with increased performance accuracy in older adults, in the more complex task condition. This ‘BOLD-performance’ relationship suggests that the neural correlates linked with successful performance in the older adults are related to specific working memory processes present in the complex but not in the baseline task condition.

There are errors in the second and third sentences of the second paragraph of the “Independent component analysis (ICA)” portion of the “fMRI image analysis” subsection of the Materials and Methods. The correct sentences are: The GLM design matrix was based on the task events (onsets), as well as the movement parameters derived from the realignment step and their first derivatives and a high pass filter of 230 seconds, implemented using a discrete cosine transform (DCT) set. The task events were convolved with three basis functions of the hemodynamic response function (HRF): the canonical HRF, its time derivative and its dispersion derivative.

There are multiple errors throughout the “Performance and age” section of the Results. The correct text is: Older adults had a mean accuracy cost of.23 (SD = .12) and a mean speed cost of.47 (.19). Younger adults had a mean accuracy cost of.08 (.04) and a mean speed cost of.31 (.18). Coefficients for the main effects of age on accuracy and speed cost were based on the regression model assessing the effects of age and/or BOLD load effect in the VLPFC component. Older adults had a higher accuracy cost (β age = .147, t(74) = 7.08, p < .0005; *R2* = .419, F(3,74) = 18.8, p < .0005) and speed cost (β age = .167, t(74) = 4.01, p < .0005; *R2* = .215, F(3,74) = 6.5, p < .0005) than younger adults. Additional regression models, performed in each load condition, revealed that older adults had lower accuracy scores in the 2-back load than younger adults (β age = −.129, t(74) = −6.47, p < .0005; *R2* = .414, F(3,74) = 16.7, p < .0005). Furthermore, older adults were slower than younger adults in both load conditions (0-back: β age = 115.32, t(74) = 9.83, p < .0005; *R2* = .581, F(3,74) = 32.9, p < .0005 and 2-back: β age = 249.01, t(74) = 9.61, p < .0005; *R2* = .569, F(3,74) = 31.3, p < .0005; see Table 2).

There are multiple errors throughout the “Components of interest: Age and BOLD load effect” section of the Results. The correct text is: The main repeated measure ANOVA on the BOLD load effects observed in the eight ICs associated with working memory processes, showed a general interaction between age and BOLD load effect (F(7,511) = 3.1, p = .007). To identify which of these 8 ICs showed an age-related BOLD load effect, additional post-hoc two sample t-tests were performed. These tests revealed that the difference in BOLD activation between the 2-back and the 0-back load condition was larger for older than younger adults in 3 ICs. Namely, the ICs containing mainly the VLPFC (t(73) = 1.9, p = .061), the right FPN (t(73) = 2.2, p = .035) and the left FPN (t(73) = 4, p < .0005).

After identifying these 3 ICs, we subsequently performed a one-sample t-test in younger and older adults, separately. The purpose of these tests was to investigate whether the BOLD activation in each of the 3 selected ICs differed significantly between the 0-back and the 2-back load condition within each age-group. The one-sample t-test was significant in all 3 ICs, for younger (VLPFC: t(37) = 6.5, p< .0005; right FPN: t(37) = 3, p = .005 and left FPN: t(37) = -4.1, p< .0005) and older adults (VLPFC (t(36) = 9.8, p< .0005), the right FPN (t(36) = 6, p< .0005 and the left FPN (t(36) = -1.7, p = .093). For all participants, the BOLD activation in the right FPN and the VLPFC increased with task load. However, the BOLD activation of the left FPN was negatively modulated by the task, as revealed by the negative beta-weights and the positive spatial map of this component (see Fig 2B and Fig 3). In young adults, the BOLD activation in the left FPN became more negative with increasing task demands. To determine whether age modulated the BOLD signal in these 3 ICs of interest in the 0-back, in the 2-back or in both load conditions, subsequent post-hoc independent two sample t-tests were performed. These tests showed that compared to younger adults, older adults had a higher BOLD activation in the VLPFC (t(73) = 3, p = .006) and the right FPN (t(73) = 2.4, p = .020), in the 2-back load condition. In the 0-back load condition, younger and older adults showed similar BOLD activation in the VLPFC and the right FPN. On the other hand, older adults had a more negative BOLD response in the left FPN than younger adults, in the 0-back load condition (t(73) = 4.5, p < .0005). Younger and older adults had comparable BOLD load effect in the other working memory related ICs.

**Fig 3:**
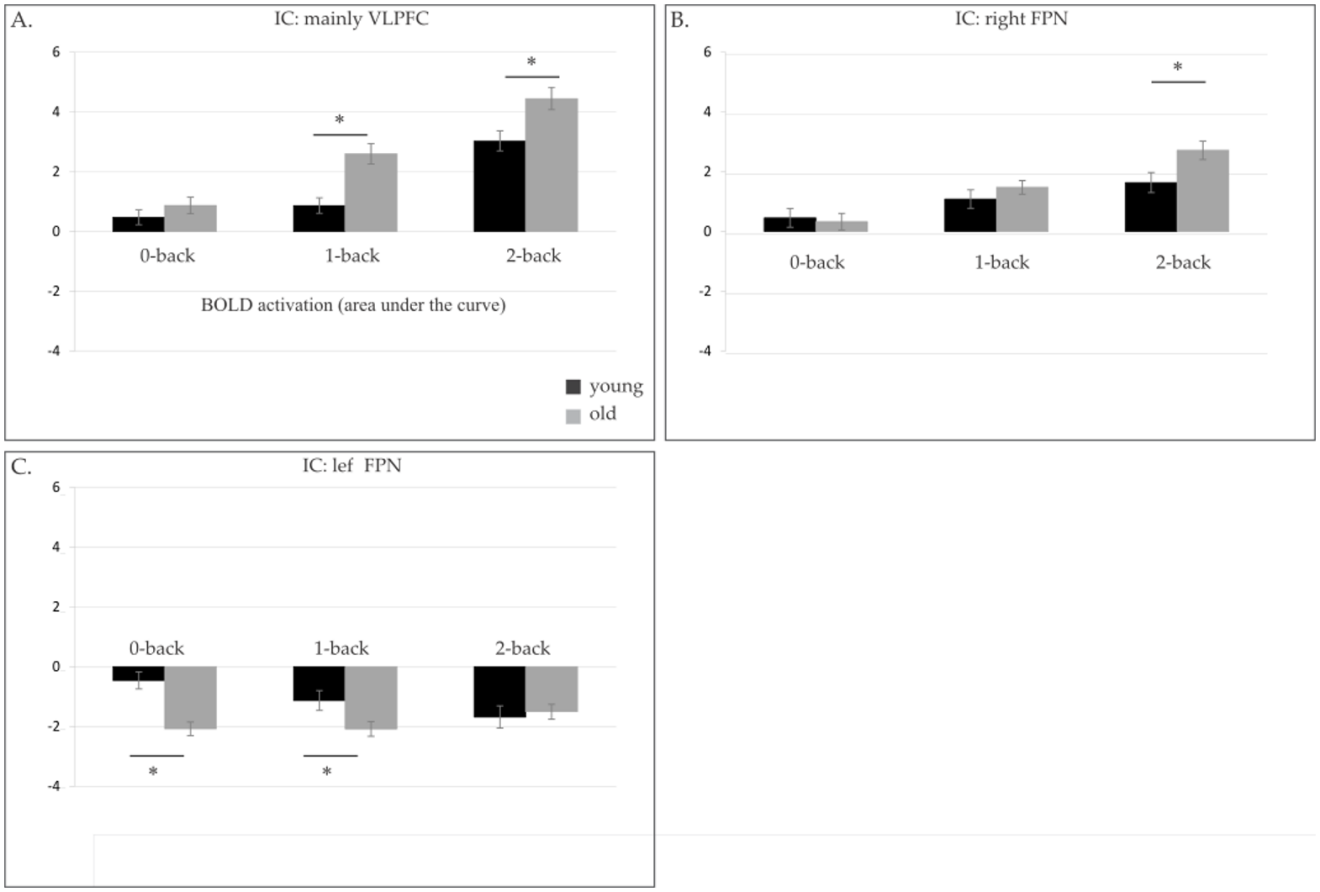
BOLD activation (area under the curve) in A) VLPFC, B) right FPN and C) left FPN, for younger (black) and older adults (grey), in the 0-back, 1-back and 2-back load conditions. Significant differences in BOLD activation between younger and older adults are noted by a star.

There are multiple errors throughout the “Performance and BOLD activation” section of the Results. The correct text is: We determined the association between behavioral outcomes (accuracy cost and speed cost) and BOLD load effect in each of the 3 ICs, in separate linear regression models (see section 2.8). The BOLD load effect in the VLPFC was significantly associated with accuracy cost and (β_load effect VLPFC_ t(74) = -2.2, p = .033). Post-hoc analysis revealed that the association between accuracy rates and BOLD activation in the two back load condition differed between the two age groups (β_age*BOLD activation VLPFC_ t(74) = 2.2, p = .034; R^2^ = .41, F(3,74) = 16.7, p < .0005), whereas there was no association between BOLD and performance in the 0-back load condition. To further investigate the association between accuracy rate and BOLD activation in the VLPFC in older adults, separate regression models were performed for the 2-back load condition, with accuracy rate as a dependent variable and the mean BOLD activation in the VLPFC component (z-scored, separately for older and younger adults) as a predictor. A larger mean BOLD activation in older adults was associated with higher accuracy rates in the 2-back load condition (β _BOLD activation VLPFC old_ t(36) = 2.12, p = .041; *R*2 = .114, F(1,36) = 4.5, p = .041; see Fig 4). The correlations between performance accuracy and BOLD activation in the VLPFC in older adults remained significant even after removal of outliers.

**Fig 4:**
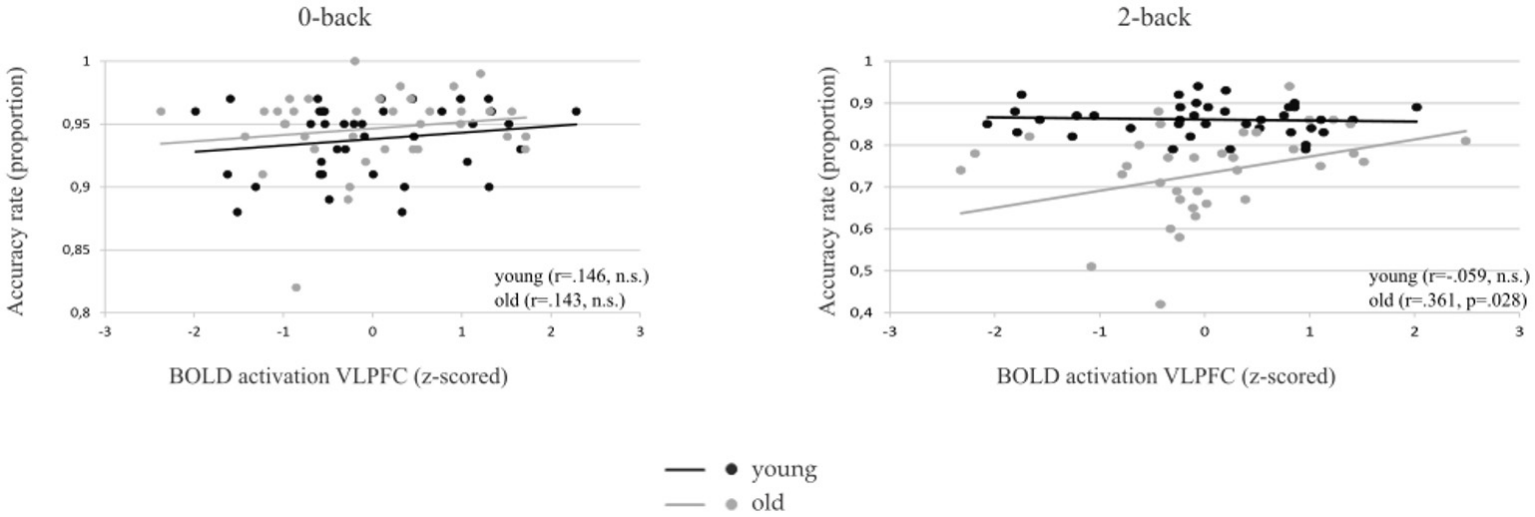
BOLD activation (z-scored) is positively correlated with accuracy rate (proportion) in the 2-back (right), but not in the 0-back (left) load condition, for older (grey) adults. There is no relationship between BOLD activation and accuracy rate in younger adults (black). Note that the axes have been scaled individually to optimize visualization.

Moreover, z-statistics [53] were used to investigate whether the correlations between accuracy scores and BOLD activity in the VLPFC differed between younger and older adults. In the 0-back load condition, the ‘accuracy-BOLD’ correlations did not differ significantly between age groups (Spearman’s rho _young_ = .146 and Spearman’s rho _old_ = .143, z = -0.01, p = .992). In the 2-back load condition, the ‘accuracy-BOLD’ correlations differed between the younger (Spearman’s rho = -.089) and the older (Spearman’s rho = .361) adults (z = -1.82, p = .034).

Moreover, a more similar BOLD response between the two load conditions in the left FPN, was associated with a smaller increase in RT in the 2-back relative to the 0-back load condition in younger adults (β _load effect left FPN younger_ t(37) = -2.24, p = .031; *R*2 = .123, F(1,37) = 5.03, p = .031; referring to β _age*BOLD load effect of the left FPN_ t(74) = -1.69, p = .094). We did not observe a significant interaction between speed cost and BOLD load effect in this IC for older adults (β load effect left FPN old t(36) = .36, n.s). The association between speed cost in younger adults and BOLD load effect in the left FPN was investigated separately for each load condition. Response speed was not associated with mean BOLD activation in either load condition.

There are multiple errors in the third paragraph of the Discussion. The correct paragraph is: In addition to the behavioural indices (i.e. speed and accuracy) we examined neural activity underlying working memory performance in both young and older individuals. In accordance with previous studies we found changes in activity patterns in brain areas implicated in working memory. Moreover, our results showed that variability in performance was related to differences in neural network activity in the older participants. The data revealed that a smaller accuracy cost in older adults was associated with a larger BOLD load effect (increase in BOLD from the 0-back to the 2-back load condition) in the component reflecting mainly the VLPFC. Such a ‘BOLD-accuracy cost’ association was observed in the complex task condition, suggesting that the effects of aging are related to specific working memory processes, such as continuous updating of working memory which are especially relevant in the 2-back condition.

There is an error in the first sentence of the fourth paragraph of the Discussion. The correct sentence is: Larger BOLD activation in the VLPFC was also associated with higher accuracy rates in the elderly in the complex task condition.

There is an error in the second sentence of the fifth paragraph of the Discussion. The correct sentence is: The positive association between accuracy rates and BOLD activation in the VLPFC in older adults is in line with these findings, and may suggest that optimal performance in the more demanding condition of our task relies on more successful maintenance of verbal information and/or successful ‘stimulus-probe’ comparisons.

The fifth sentence of the sixth paragraph of the Discussion should be deleted. The relevant sentence is: Cognitive control is engaged during both simple and more complex task conditions [61].

There is an error in the last sentence of the seventh paragraph of the Discussion. The correct sentence is: In addition, the presence of this relationship in the more but not in the less demanding task condition suggests that this compensatory neural mechanism is related to sub-processes within the working memory system.

There is an error in the second to last sentence of the last paragraph of the Discussion. The correct sentence is: This ‘BOLD-performance’ relationship was observed in the complex task conditions, supporting that the neural mechanisms underlying performance in older adults are related to working memory processes such as manipulation or updating of information.

The image for Fig 3 is incorrect. Please see the corrected Fig 3 here.

The image and caption for Fig 4 are incorrect. Please see the complete, corrected Fig 4 here.
